# Supplementary figures and images for: An integrative network-based approach for drug target indication expansion
Source: PLoS One. 2021 Jul 9;16(7):e0253614. doi: 10.1371/journal.pone.0253614 (PMC8270215; doi:10.1371/journal.pone.0253614)

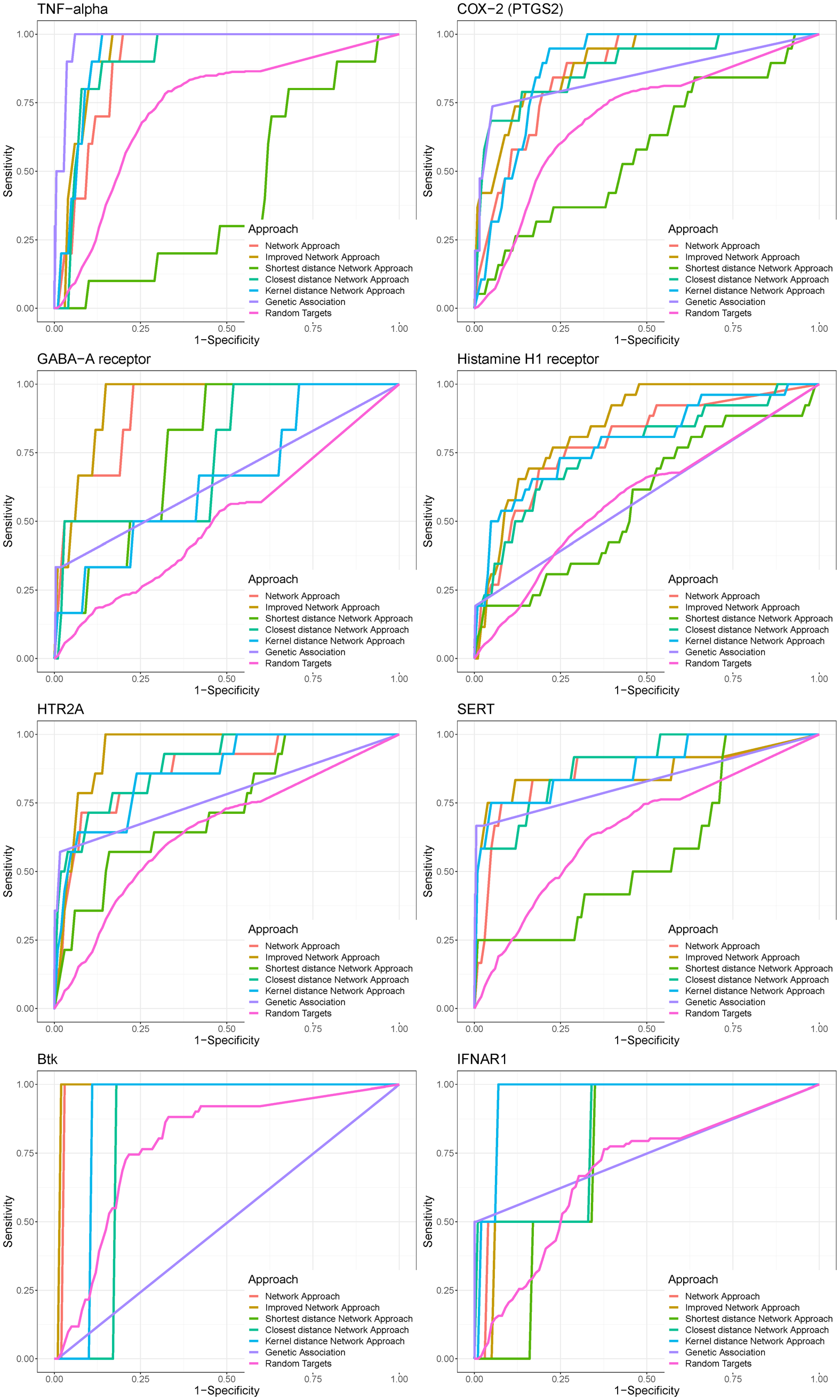

Supplement: S1 Fig — (TIF) [file pone.0253614.s001.tif]

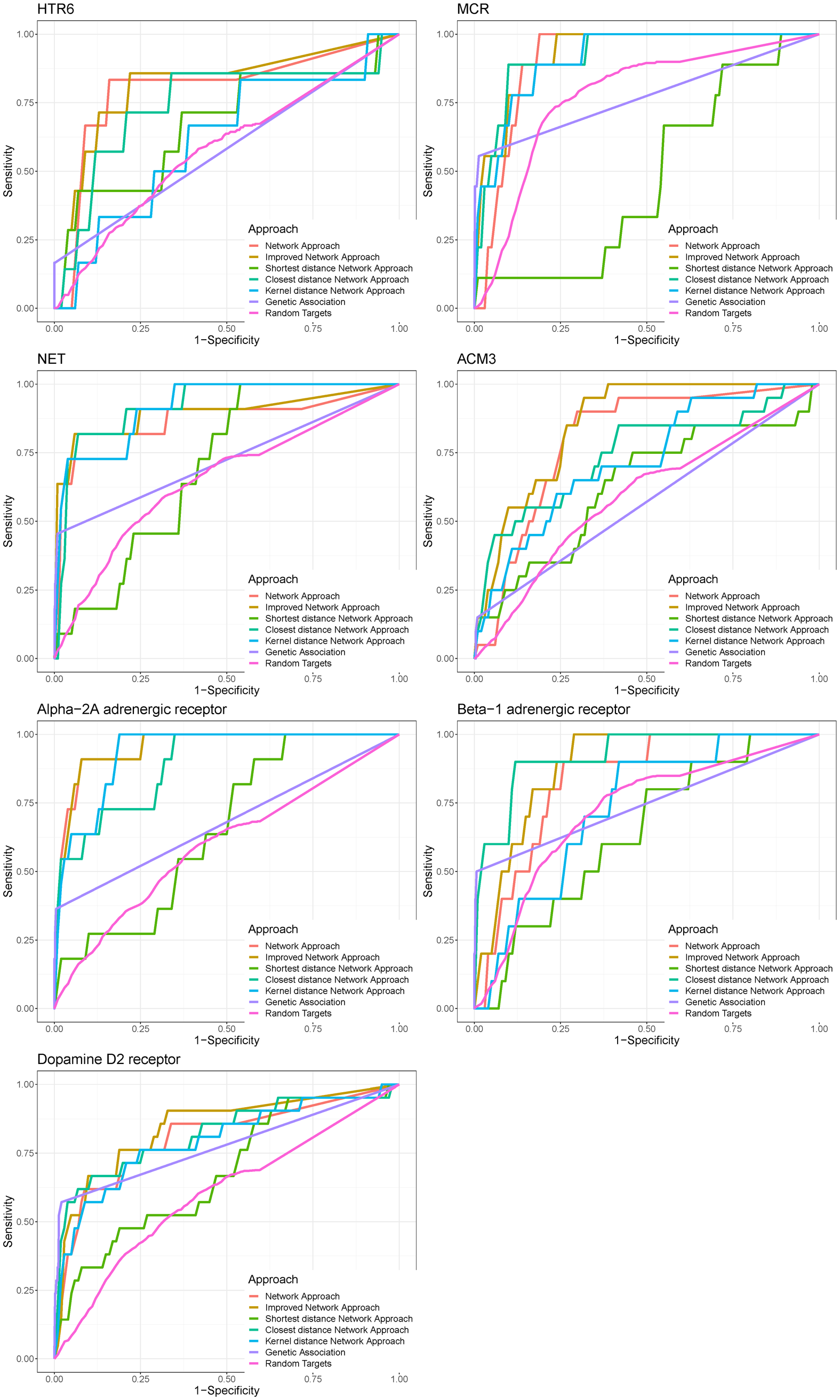

Supplement: S2 Fig — (TIF) [file pone.0253614.s002.tif]
